# A geographic distribution database of *Mononychellus* mites (Acari, Tetranychidae) on cassava (*Manihot esculenta*)

**DOI:** 10.3897/zookeys.407.7564

**Published:** 2014-05-08

**Authors:** Aymer Andrés Vásquez-Ordóñez, Soroush Parsa

**Affiliations:** 1 CIAT, Centro Internacional de Agricultura Tropical (CIAT), Apartado Aéreo, 6713 Cali, Colombia

**Keywords:** Cassava Green Mite, Cassava Green Mite Complex, International Center for Tropical Agriculture (CIAT), CIAT’s Arthropod Reference Collection (CIATARC)

## Abstract

The genus *Mononychellus* is represented by 28 herbivorous mites. Some of them are notorious pests of cassava (*Manihot esculenta* Crantz), a primary food crop in the tropics. With the exception of *Mononychellus tanajoa* (Bondar), their geographic distribution is not widely known. This article therefore reports observational and specimen-based occurrence data of *Mononychellus* species associated with cassava. The dataset consists of 1,513 distribution records documented by the International Center for Tropical Agriculture (CIAT) between 1975 and 2012. The specimens are held at CIAT’s Arthropod Reference Collection (CIATARC). Most of the records are from the genus’ native range in South America and were documented between 1980 and 2000. Approximately 61% of the records belong to *M. tanajoa*, 25% to *M. caribbeanae* (McGregor), 10% to *M. mcgregori* (Flechtmann and Baker) and 2% to *M. planki* (McGregor). The complete dataset is available in Darwin Core Archive format via the Global Biodiversity Information Facility (GBIF).

## Data published through GBIF

http://www.gbif.org/dataset/785cf038-7b79-4c2f-9e9e-eb940fcd4c0c

## Project details

**Project title:** Management of RTB Critical Pests and Diseases under Changing Climates, through Risk Assessment, Surveillance and Modeling

**Project personnel:** Aymer Andrés Vásquez-Ordóñez (Data Manager, Data Publisher), Rodrigo Zúñiga (Data Manager), Soroush Parsa (Principal Investigator, Data Publisher).

***Mononychellus* collectors:** Collectors who have deposited more than 50 specimens include: Julio Bonilla, Daniel González, José María Guerrero, Carlos Julio Herrera, Jorge Ivan Lenis, Nora Cristina Mesa, Jesús Antonio Reyes, César Rodríguez and Miguel Santiago Serrano.

**Funding:** This project was supported by the Roots, Tubers and Bananas (RTB) Research Program of the Consultative Group on International Agricultural Research (CGIAR).

**Design description:** The purpose of this dataset is to significantly increase the geographic distribution data publicly available for the genus *Mononychellus*. This genus includes several species of herbivorous mites that are major pests of cassava (*Manihot esculenta* Crantz), most notoriously *Mononychellus tanajoa* (Bondar). We report 1,513 distribution records of the genus, documented by the International Center for Tropical Agriculture (CIAT) between 1975 and 2012. Most of the records (53%) correspond to specimens preserved at CIAT’s Arthropod Reference Collection (CIATARC). Prior to this contribution, only 30 distribution records of *Mononychellus* were accessible through the Global Biodiversity Information Facility (GBIF) data portal (accessed 1/13/2014). Accordingly, the CIATARC *Mononychellus* dataset should facilitate a much better understanding of the genus’ geographic association with cassava.

## Taxonomic coverage

**General taxonomic coverage description:** Most records were identified to species level (98%) with the help of expert input (José María Guerrero, Pilar Hernandez). Only four species of the genus are reported. Approximately 61% of the records belong to *Mononychellus tanajoa*, 25% to *Mononychellus caribbeanae* (McGregor), 10% to *Mononychellus mcgregori* (Flechtmann and Baker) and 2% to *Mononychellus planki* (McGregor).

## Taxonomic ranks

**Kingdom:** Animalia.

**Phylum:** Arthropoda.

**Class:** Arachnida.

**Order:** Trombidiformes.

**Family:** Tetranychidae.

**Genus:** *Mononychellus*.

**Species:** *Mononychellus caribbeanae*, *Mononychellus mcgregori*, *Mononychellus planki*, *Mononychellus tanajoa*.

**Common name:** Cassava Green Mite (for *Mononychellus tanajoa*), Cassava Green Mite Complex (for *Mononychellus caribbeanae*, *Mononychellus mcgregori*, *Mononychellus planki* and *Mononychellus tanajoa*)

## Spatial coverage

**General spatial coverage:** The *Mononychellus* specimens and observations of CIATARC are from South America (14 countries) and Central America (Cuba, Haiti, Honduras, Mexico, Nicaragua, Trinidad and Tobago), which represent the 99% of records, with Colombia and Venezuela are the best represented countries, followed by Brazil and Ecuador (Fig. 2). These countries are considered the center of origin of our focal species. The remaining records belong to Africa (0.6%; Benin, Kenia, Mozambique, Nigeria) and Asia (0.3%; Vietnam, China).

**Coordinates:** 22.904301 and -27.098576 latitude; -95.2174947 and 109.580811 longitude.

**Figure 1.**
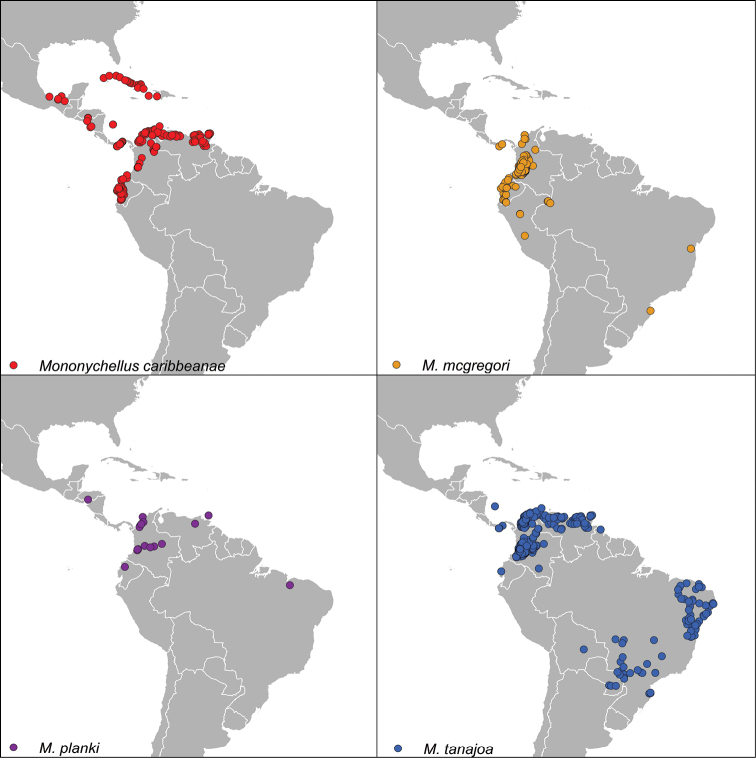
Native geographic distribution of records of the CIATARC *Mononychellus* dataset in the American continent.

**Figure 2.**
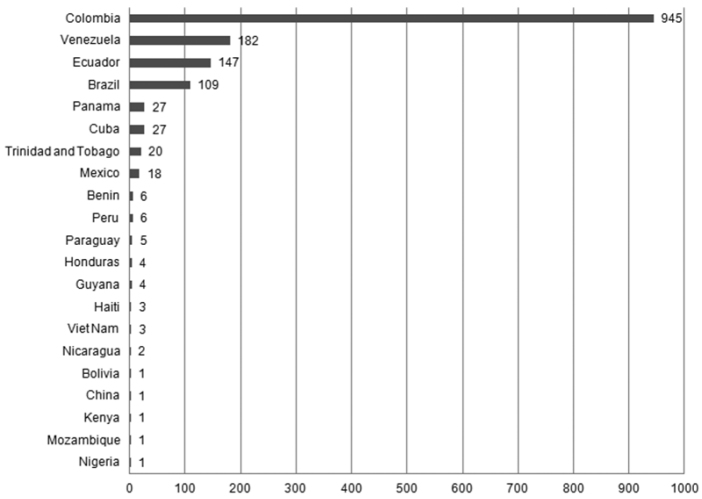
Records by country in the CIATARC *Mononychellus* dataset.

## Temporal coverage

1975–2012.

## Natural Collections descriptions

**Collection name:** CIAT Arthropod Reference Collection (CIATARC).

**Specimen preservation method:** Specimens are preserved as microslide preparations in microscope slide boxes within cabinet drawers maintained at 21.0 ± 0.4 C and 47.6 ± 8.6 relative humidity. They are sorted numerically by species and country of origin.

**Curatorial unit:** 3,510 with an uncertainty of 0 (microslide preparation).

## Methods

**Method step description:** The dataset integrates two data flows: observational records and specimen-based records, identified either to genus or to species. The former were digitized from field diagnostic forms completed by personnel extensively trained in mite identification. These identifications, however, were likely conducted on site without mounting and preserving samples. Alternatively, these observations may correspond to properly-mounted but lost specimens. In either case, our confidence in the identification of observational records is high to the genus level, but moderate to the species level. On the other hand, specimen-based records belong to verifiable samples properly-preserved at CIATARC following the guidelines of Krantz (1978). Unique accession numbers were assigned to all records.

All biodiversity data available (i.e. specimen, species identification, name of determiner, sex, biological phase, locality, date, habitat, host, collector and observations) was digitized in a Microsoft Excel 2010 spreadsheet adopting the Darwin Core Archive format v1.2 (Wieczorek et al. 2012). We updated locality fields (e.g., district, municipality) using the most current names and classifications of administrative divisions used by each country (e.g. http://www.dane.gov.co/Divipola/ for Colombia, http://www.inec.gob.ec/estadisticas/?option=com_content&view=article&id=80 for Ecuador, etc. [accessed 2013/11/14]). Based on their locality names, we then geocoded the records using Google Maps (https://maps.google.com/), GeoNames (http://www.geonames.org/) or Amézquita et al. (2013). GPS coordinates were converted to decimal degrees. The dataset with metadata was uploaded to the Integrated Publishing Toolkit (IPT) of the Colombia node of Global Biodiversity Information Facility (GBIF) (http://www.gbif.org/dataset/785cf038-7b79-4c2f-9e9e-eb940fcd4c0c).

**Sampling description:** The records in the dataset have been documented in three ways:

1) Records from CIAT’s initial field explorations to document pests in cassava (Guerrero and Bellotti 1981; 4.4% records, between 1975-1983).

2) Records documented during the “Cassava Green Spider Mite Biological Control Project,” led by CIAT, International Institute of Tropical Agriculture (IITA), Commonwealth Institute of Biological Control (CIBC) and Empresa Brasileira de Pesquisa Agropecuária (EMBRAPA) (Bellotti et al. 1987, 1996, 1998, 2000, Byrne et al. 1983, Braun et al. 1993, CIAT 1984, 1985, 1986, 1990, 1992, 1993, 1995, Guerrero et al. 1993, CIAT et al. 1998; 89.6%, 1983–1999). Their locations were systematically selected based on their climatic homology to *Mononychellus tanajoa*-affected areas in Africa (Bellotti et al. 1987, CIAT 1993, Guerrero et al. 1993).

3) Records from other sources; including field inspections and collections conducted during routine farm visits by CIAT personnel, and from specimens submitted to CIATARC by fellow institutions and researchers (Bellotti et al. 2000; CIAT 2001, 2002, 2003; 6%, 2000-2012).

The sampling process typically involved scouting cassava fields for infested plants, identified by speckling of their terminal leaves, followed by a close-up inspection for green mites using a 10× magnifying glass. To collect specimens, mites were then brushed off from leaves into collection vials containing a lactophenol solution (Krantz 1978) and maintained in ice chests until reaching the laboratory for proper mounting and identification (Bellotti et al. 1987, CIAT 1993, Guerrero et al. 1993).

**Quality control description:** Record validation and cleaning was incorporated at several steps of the documentation process, following guideless by Chapman (2005a, b). The scientific names on labels were checked with a taxonomic thesaurus developed by AAV. This thesaurus compiled all known synonyms and spelling variants of the scientific names used for our focal species. We assigned scientific names in accordance to current taxonomy trends. Geographic coordinates were verified using the “Check Coordinates” function in DIVA-GIS (Hitmans et al. 2001). For this last step, we relied on the Global Administrative Unit Layers (GAUL) shape file developed by the Food and Agriculture Organization of the United Nations (FAO, http://www.fao.org/geonetwork/srv/en/metadata.show?id=12691, [accessed 2013/11/14]).

## Datasets

### Dataset description

**Object name:** Darwin Core Archive *Mononychellus* distribution: data of the CIAT Arthropod Reference Collection of International Center for Tropical Agriculture (CIAT).

**Character encoding:** UTF-8.

**Format name:** Darwin Core Archive format.

**Format version:** 1.0.

**Distribution:** http://www.gbif.org/dataset/785cf038-7b79-4c2f-9e9e-eb940fcd4c0c

**Publication date of data:** 2014-03-14.

**Language:** English.

**Licenses of use:** This dataset [*Mononychellus* Collection of CIAT Arthropod Reference Collection (CIATARC)] is made available under the Creative Commons Zero (CC0) 1.0.
